# Does Serum Urate Change as Parkinson’s Disease Progresses?

**DOI:** 10.3233/JPD-202064

**Published:** 2020-10-27

**Authors:** Yasemin G. Hasimoglu, Xiqun Chen, Rachit Bakshi, Michael A. Schwarzschild, Eric A. Macklin

**Affiliations:** a Department of Neurology, MassGeneral Institute for Neurodegenerative Disease, Massachusetts General Hospital, Boston, MA, USA; b Department of Medicine, Biostatistics Center, Massachusetts General Hospital, Boston, MA, USA

**Keywords:** Parkinson’s disease, urate, disease progression, longitudinal study

## Abstract

Higher serum urate concentration is associated with decreased risk of Parkinson’s disease (PD) as well as slower disease progression, but its relationship with severity of PD remains unclear. This study investigated whether changes in serum urate concentration over 5 years were associated with disease progression assessed by MDS-UPDRS Part III score, Hoehn and Yahr stage, or DaTscan imaging. Average serum urate concentration was stable over time and change in serum urate concentration did not correlate with worsening of measures of PD progression. These results suggest that serum urate concentration is not a monitoring biomarker of PD progression in early stages.

## INTRODUCTION

Parkinson’s disease (PD) is a neurodegenerative disease characterized by loss of dopaminergic nigrostriatal neurons and progressive motor and cognitive deficits. Although the pathogenic mechanisms underlying the disease remain unclear, increasing evidence suggests that oxidative stress plays a critical role in the neurodegenerative process [1]. Higher levels of urate, an endogenous antioxidant, represents an established biomarker of reduced risk of PD and of a slower rate of its progression [2]. The possibility that urate may also be a marker of disease severity has been suggested by cross-sectional analyses of PD patients linking lower serum urate levels to more advanced disease state assessed by Hoehn and Yahr (H & Y) stage or Unified Parkinson’s Disease Rating Scale (UPDRS) score [3–6]. However, rather than reflecting changes in serum urate as PD patients progress, the apparent association between lower serum urate and more advanced disease state could also result from an overrepresentation of patients with constitutively lower urate levels and faster progression rate among patients with later stage PD. Analysis of longitudinal data is required to determine whether urate concentration changes in association with the progression and increasing severity of the disease. Our study aimed to correlate longitudinal changes in disease severity with longitudinal changes in serum urate concentration in a well-characterized cohort of PD patients to clarify whether urate may serve as a monitoring biomarker of PD stage as well as a predictive biomarker of PD risk and rate of progression.

## METHODS

Subject data from the *de novo* PD and healthy control (HC) cohorts were obtained from the Parkinson’s Progression Markers Initiative (PPMI) database (http://www.ppmi-info.org/data). For up-to-date information on the study, visit http://www.ppmiinfo.org. The PD cohort comprised 423 subjects who received a PD diagnosis two or fewer years prior to enrollment, who had not taken PD medication, and whose dopamine transporter (DAT) ligand (DaTscan™) brain scans demonstrated DAT deficit consistent with PD at baseline. The HC cohort consisted of 196 subjects. Disease severity was characterized in 3 ways: nigrostriatal dopamine receptor density estimated by DaTscan imaging, Movement Disorders Society-sponsored revision of the Unified Parkinson’s Disease Rating Scale (MDS-UPDRS) Part III score, and Hoehn and Yahr (H & Y) stage. Disease severity from DaTscans was quantified in two ways, as the striatal binding ratio (SBR) obtained from the ipsilateral putamen alone and as the average of SBRs obtained from the caudate and putamen in both hemispheres. DaTscans were assessed at baseline for both PD and HC participants and at year 1, year 2, and year 4 of the study for PD participants. Urate concentrations, MDS-UPDRS scores and H & Y stages were assessed annually. If subjects initiated PD medication after baseline, MDS-UPDRS scores and Hoehn & Yahr stages assessed in the “off” state, evaluated at least 6 hours after last dose of antiparkinsonian medication, were included in the analysis.

Change during 5-year follow-up in serum urate, DaTscan SBR, MDS-UPDRS Part III and H&Y stage for both PD and HC subjects was estimated from separate linear mixed effects (LME) models, each adjusted for baseline age, sex, and time-dependent body mass index (BMI). In the models for MDS-UPDRS score and H&Y stage, time since baseline; baseline age, sex, BMI and their two-way interactions with time since baseline; group (PD or HC), and its two-way interactions with age, sex, and BMI; and their three-way interactions with time were included as fixed terms. The MDS-UPDRS model additionally included a time-dependent levodopa-equivalent daily dosage (LEDD) term. The model for DaTscan SBR was similar to the model for H & Y, but included no two- or three-way interactions with group given lack of follow-up among HC participants. An intercept and slope over time were included as correlated random effects varying by subject in each model. Least-square mean estimates of adjusted urate, DaTscan SBR, MDS-UPDRS Part III scores and H&Y stages were calculated for each time point using mean values for baseline age, BMI, and male sex percentage.

The correlation between change in serum urate and change in each of the three disease progression markers was estimated for changes from baseline to year 4 after adjusting for potential confounders. Four-year change in ipsilateral putamen DaTscan SBR, MDS-UPDRS Part III score, and H&Y stage were modeled in separate multiple regressions adjusting for baseline age, baseline BMI, 4-year change in BMI, baseline urate and baseline progression measure. The change in MDS-UPDRS Part III scores additionally adjusted for baseline LEDD and 4-year change in LEDD. Similarly, four-year change in serum urate concentration was modeled in separate multiple regressions adjusting for corresponding covariates: baseline age, baseline BMI, 4-year change in BMI, baseline urate and baseline progression measure, one model for each progression measure. Residuals after adjustment for a given progression marker and for serum urate were compared by Pearson correlation. This two-step approach is equivalent to a larger multiple regression model regressing four-year change in a given measure on four-year change in serum urate with the stated adjustments but allows us to express the relationships as partial correlations rather than as regression coefficients [7]. Each correlation was obtained from all subjects that had baseline and year 4 data available for urate and the PD progression marker in question (*n* = 257, 211, and 211 for correlation with DaTscan, MDS-UPDRS Part III score, and H & Y stage, respectively).

## RESULTS

**Fig. 1.**
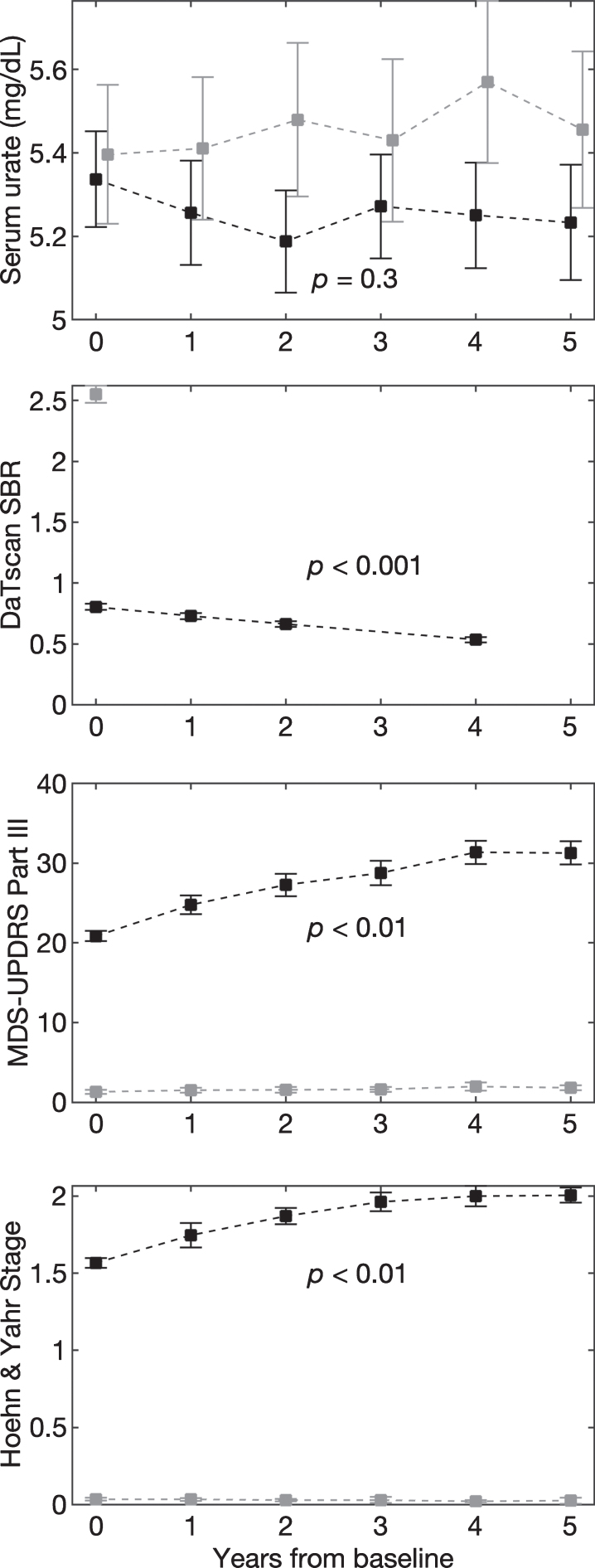
Age-, sex-, and BMI-adjusted mean values of urate (A), DaTscan SBR from ipsilateral putamen (B), MDS-UPDRS Part III score (C), and Hoehn & Yahr stage (D) over 5 years. Black and grey dashed lines represent PD and healthy control subjects, respectively. Error bars indicate 95% confidence intervals. *p*-values are shown for the trend over time for PD subjects.

**Fig. 2.**
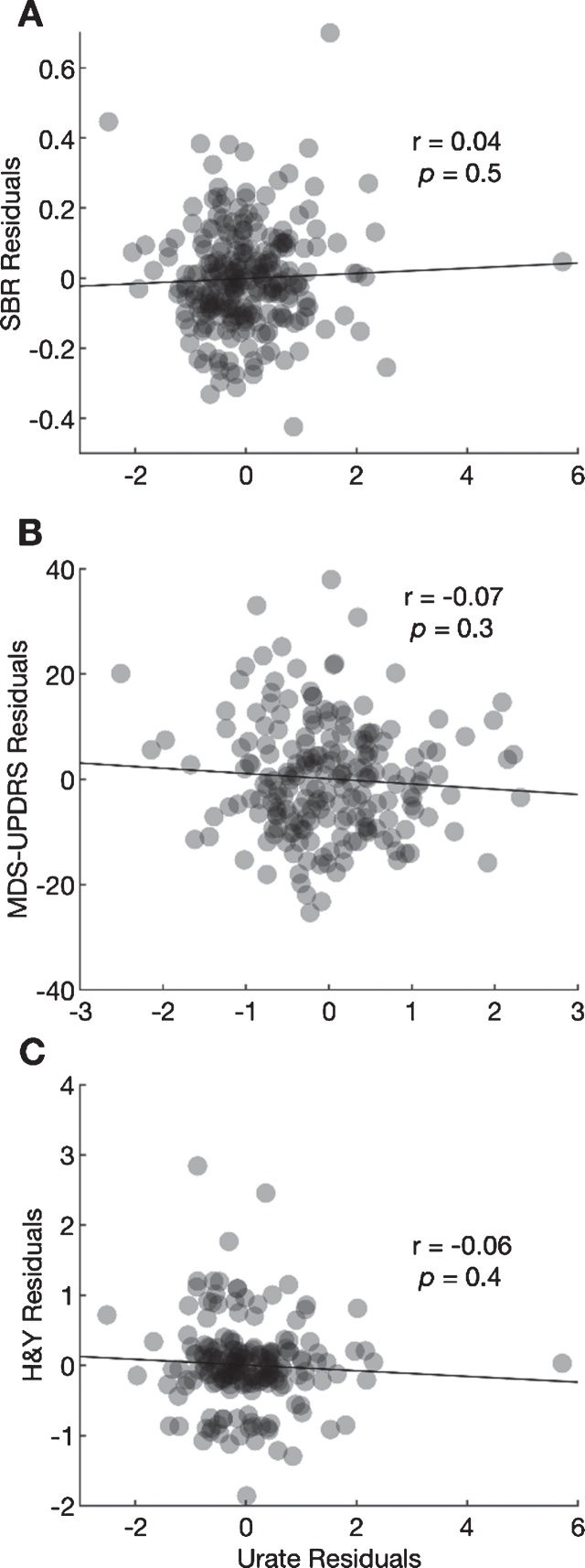
Scatter plots between the residuals from a regression of 4-year change in ipsilateral putamen SBR (A), MDS-UPDRS Part III score (B), and H&Y stage (C) and residuals from regressions of 4-year change in urate. Pearson coefficients and their *p*-values are shown for each correlation.

Baseline characteristics of PD and HC subjects were comparable for age, sex, BMI, and serum urate (Table 1). Mean urate concentrations of PD subjects, were stable over time both before (data not shown), and after adjustment (Fig. 1A; –0.006 mg/dL/yr, 95% CI –0.020 to 0.005 mg/dL/yr, *p* = 0.32). Mean urate concentrations of matched HC subjects were similarly stable (Fig. 1A; 0.01 mg/dL/yr, 95% CI –0.03 to 0.020 mg/dL/yr, *p* = 0.19), however, their absolute levels were slightly but significantly higher on average than levels among DAT-deficient early PD subjects (mean±SE: 5.26±0.03 mg/dL vs. 5.45±0.04, *p* < 0.001). Established radiographic and clinical measures of PD severity progressively changed as expected in this PD cohort (Fig. 1B–D). Mean adjusted DaTscan SBR from the ipsilateral putamen decreased (Fig. 2B; –0.033 units/yr, 95% CI –0.035 to –0.032 units/yr, *p* < 0.001), as did the average SBR from the whole striatum (–0.048 units/yr, 95% CI –0.050 to –0.46 units/yr, *p* < 0.001). Mean MDS-UPDRS Part III score (1.05 units/yr, 95% CI 0.77 to 1.32 units/yr, *p* = 0.002) and H & Y stage (0.04 units/yr, 95% CI 0.03 to 0.06 units/yr, *p* = 0.005) increased. The same trends were also present without adjustment (data not shown).

**Table 1:** Baseline characteristics of study participants

	PD (*n* = 423)		HC (*n* = 196)	
	Mean	SD	Mean	SD
Age	61.7	9.7	60.8	11.2
Male (%)	65	–	64	–
BMI	27.1	4.6	26.9	4.4
Serum urate (mg/dL)	5.3	1.3	5.4	1.3
DaTscan SBR	1.4	0.4	2.6	0.6
MDS-UPDRS Part III score	20.9	8.6	1.2	2.2
Hoehn &Yahr stage	1.6	0.5	0.02	0.2

The Pearson correlation between the residual change in urate and the residual change in the ipsilateral putamen SBR after adjustment for potential confounders was 0.04 (95% CI –0.08 to 0.16; Fig. 2A, *p* = 0.49). The correlation between the residual change in urate and residual change in MDS-UPDRS Part III scores was –0.07 (95% CI –0.21 to 0.30; Fig. 2B, *p* = 0.30), and the correlation with residual change in H & Y stage was –0.06 (95% CI –0.20 to 0.08; Fig. 2C, *p* = 0.41). None of the correlations was large or significant.

## DISCUSSION

The results of this longitudinal study demonstrate that serum urate concentrations do not track increasing severity of PD as patients progress in early stages of the disease. Previous analyses of PPMI data reported no significant difference in urate concentrations between PD and HC subjects at baseline [8] or over time, although previous analyses did not adjust for key urate covariates of age [9], sex [9], or BMI [8, 9]. Our study found the same result at baseline despite adjusting for each of these variables. However, in line with previous reports in most other cohorts [10], a comparison between PD and HC groups averaged across all time points showed that the PD group had slightly but significantly lower urate concentrations. Urate concentrations across 5 years did not trend significantly up or down for either group. This result was also replicated in an independent cohort of similarly early PD subjects in whom serum urate concentrations remained stable over more than four years [11].

Other measures of PD significantly changed with increasing time from diagnosis, consistent with the broad literature on PD severity [12]. Severity was described both with a pathophysiological marker of dopaminergic nerve terminal integrity using DaTscan SBRs, and with markers of parkinsonian motor deficits using MDS-UPDRS Part III scores and Hoehn & Yahr stages. DaTscans present a quantitative measure of *in vivo* dopamine transporter density and activity that are gradually lost in PD and can be used to assess disease progression and severity [13]. While MDS-UPDRS scores and Hoehn & Yahr stages can be influenced by antiparkinsonian treatments, the *in vivo* information provided by DaTscans is relatively resistant to such effects and is thus an appealing diagnostic to objectively and quantitatively describe PD severity. However, motor assessments of the disease are often more straightforward to interpret and more directly relate to patient experience. As such, they are relevant indicators of the manifestation of PD, despite their greater susceptibility to confounders. The progression of each of these measures over time (Fig. 1B–D) illustrates the progression of disease severity of PD in its own way. Overall, none of these characterizations of PD progression correlated with individual changes in serum urate concentrations over time after adjustment for confounders. These results suggest that serum urate concentration is not a monitoring biomarker for the clinical state or severity of PD, and thus its serial measurement cannot contribute as a clinical tool for tracking the disease.

In contrast to our study of longitudinal severity data, previous studies reporting an inverse relationship between serum urate to PD severity were cross-sectional in design [3–6]. Accordingly, they may be confounded by differences between early- and late-stage subjects other than time. For example, the more advanced PD participant group may be enriched for individuals who have progressed more quickly, or who have lost more weight, and accordingly lowered their BMI, both features associated with lower urate concentrations [2, 14, 15]. None of these studies adjusted for serum urate at diagnosis or for BMI, which could have biased their results. Our study accounted for the rates of change of both urate and PD as well as baseline urate levels. Additionally, our analyses adjusted for BMI in addition to age and sex, and consequently also eliminated any changes in urate that could have occurred due to changes in BMI over time.

Limitations of our study include the reliance of our assessments of progression on absolute 4-year changes, which limited our analysis to those subjects who had 4-year follow-up data for all variables of interest. Our analysis is also limited by a focus on a small number of measures of PD progression related to motor function and pathways. We cannot rule out associations between change in urate concentration and change in non-motor features of PD progression, which are not captured by DaTscan SBR, MDS-UPDRS Part III, or H & Y stage. We also cannot rule out small but meaningful associations between changes in urate concentration and changes in PD progression given the size of the available sample and resulting precision of our estimates. False negative findings are possible.

Additionally, genetic PD sub-populations were not excluded from our main analysis of the *de novo* PD cohort of PPMI, of whom 16% carry mutations of *LRRK2* or *GBA*, two genes that confer substantial risk of developing PD depending on the specific genotype [17]. Although it is possible that the association between urate and disease progression differs in these genetic forms, they can share similar urate associations with idiopathic PD [18]. Moreover, analyses of the PPMI *de novo* cohort that are inclusive of these carriers may be more clinically relevant to people with typical early PD whose genotype is not routinely known.

Finally, our study is based on a relatively short follow-up period available for analysis. As disability progresses steadily over a clinical course that typically runs over ten years [19], follow-up over only four years in early, initially *de novo* PD, precludes generalization to later, more severe stages of the disease. Nevertheless, in this study we present the first longitudinal analysis of the relationship between change in urate concentrations over time and increasing PD severity. Our results show that change in serum urate concentrations are not correlated with change in early PD progression and, thus, do not serve as a reliable marker of progressive disease severity.

## CONFLICT OF INTEREST

EAM served as a Data and Safety Monitoring Board (DSMB) member for Novartis Pharmaceuticals and Shire Human Genetic Therapies, served on an advisory committee for Biogen, consulted for Cerevance, Intrance, Inventram, Lavin Consulting, and Myolex, and his institution received grants on his behalf from Amylyx Pharmaceuticals, GlaxoSmithKline, and Mitsubishi Tanabe Pharmaceuticals. MAS served as a DSMB member for Eli Lilly and Company, and served on advisory boards for Denali Therapeutics, nQ Medical, and Prevail Therapeutics. Other authors report no relevant conflicts of interest.
